# A Case of Ciguatera Fish Poisoning in South Georgia

**DOI:** 10.7759/cureus.22728

**Published:** 2022-02-28

**Authors:** Pankaj Agrawal, John Grab

**Affiliations:** 1 Hospital Medicine, South Georgia Medical Center, Valdosta, USA; 2 Hospital Medicine, Edward Via College of Osteopathic Medicine, Auburn, USA

**Keywords:** mannitol, poisoning, fish, ciguatera, ciguatoxin

## Abstract

Ciguatera fish poisoning (CFP) is a seafood-associated non-infectious condition, caused by Ciguatoxins. It is known to be the most frequently reported cause of seafood-toxin-related illness in the world. CFP can cause a wide range of gastrointestinal, neurological, and cardiovascular symptoms which can last from a few days to a few months. We describe a case of ciguatera fish poisoning (CFP) in South Georgia after ingestion of a fish from the Caribbean Sea. This case report brings the attention of clinicians for early recognition of the condition and appropriate treatment.

## Introduction

Ciguatera fish poisoning (CFP) is a seafood-associated non-infectious condition, caused by Ciguatoxins. It is known to be the most frequently reported cause of seafood-toxin-related illness in the world [1]. The presence of gastrointestinal symptoms in a person soon after consuming fish along with neurological symptoms should raise suspicion of CFP. There are no definite diagnostic tests available at this time, a detailed history remains a prime diagnostic tool. This clinical condition is mainly treated with supportive care and intravenous (IV) mannitol may offer additional benefits. Early treatment with IV mannitol can help minimize the severity and duration of the illness. We discuss a case of CFP in South Georgia who was given IV Mannitol. With the globalization of the supply chain and seafood market, CFP is not limited to endemic areas only and can be seen in any part of the world.

## Case presentation

A 53-year-old Caucasian female with a past medical history of hypothyroidism came to the hospital emergency room about 36 hours after ingestion of a Grouper fish that her family has brought over from the Bahamas. She started feeling sick soon after ingestion. Her symptoms included: nausea, vomiting, abdominal cramping, and diarrhea. She also reported paresthesia in her hands. On initial evaluation, she appeared dehydrated and her vitals were in the normal range. Laboratory evaluation confirmed hemoconcentration with hemoglobin of 15.7. Other significant findings included: potassium 3.0 mmol/L, bicarb 19 mmol/L, anion gap 17 mmol/L, and aspartate transaminase (AST) 81 U/L, alanine transaminase (ALT) 76 U/L, lactic acid 4.6 mmol/L. The rest of the electrolytes were within the normal limit. The patient was given two liters of crystalloid fluid and was discharged home with ondansetron and gabapentin. The patient returns to the primary care physician’s office two days later with ongoing symptoms of nausea, vomiting, diarrhea, and fatigue. She also reported a variety of neurological symptoms including itching throughout the body without rash, numbness, and tingling in her arm, face, tongue, and other parts of the body. In addition, she reported a reversal of temperature sensations (hot things feel cold and cold things feel hot). She was admitted to the hospital for further evaluation and treatment. On examination, she appeared mildly dehydrated and afebrile with vitals in the normal range. Laboratory evaluation showed improvement from two days ago except potassium was still at 3.2 mmol/L. After correction of potassium, she was given mannitol 1 mg/kg infusion over 30 minutes. The patient reported mild worsening of her paresthesia, which was thought to be due to the release of toxins from fat cells. About 24 hours later another dose of mannitol was infused, the patient reported improvement in symptoms, especially in a rash; however, there was not a complete resolution of symptoms noted. Most data suggest that mannitol is helpful within 48-82 hours of ingestion of the fish. Our patient received the first dose of mannitol over 92 hours after ingestion. The patient had prolonged symptoms with intermittent flares even after eight months of exposure.

## Discussion

Ciguatera fish poisoning (CFP) remains the most common cause of seafood-related illness in the world. As per the Food and Agriculture Organization of the United Nations, the worldwide annual incidence of CFP has been estimated at about 50,000 cases per year and it constitutes a global health problem. Hawaii, Puerto Rico, and Florida are the mainly affected locations in the US [2]. This disease has been very underreported across the world. An analysis was done in the state of Florida during the years 2007-2011. The results showed a crude annual reporting of 0.2 cases per 100,000 population of CFP to the Florida Department of Health (FDOH). After adjustment for underreporting, the total annual incidence of CFP was estimated to be 5.6 cases per 100,000 population in the state of Florida [3].

CFP can be seen in any part of the world where fish derived from tropical reef ecosystems are being consumed. Ciguatoxin is a fat-soluble compound produced by Gambierdiscus toxicus, a type of dinoflagellate. It gets concentrated in the food chain as alga are eaten by small herbivorous fishes and subsequently these fishes are eaten by large carnivorous fishes, and eventually by humans. This is endemic in the tropical Indo-Pacific Islands and the Caribbeans [4]. The toxin is not affected by heat; thus, cooking does not protect against this illness [5]. Examples of common ciguatoxin fishes include Moray Eel, Barracuda, Grouper, Jacks, Amberjack, Snapper, Surgeonfish, Parrotfish, Wrasses, etc. [6]. In a French Polynesian study, it was estimated that health-related cost due to CFP is between $749-$1613 for each reported and unreported case [7].

Symptomatology

Most patients with CFP poisoning report gastrointestinal symptoms (nausea, vomiting, diarrhea, abdominal pain) usually within 6-12 hours of ingestion. Neurological symptoms start 1-2 days after exposure and include numbness, tingling, metallic taste, itching, muscle pain, joint pain, reversal of temperature sensation, headache, and dizziness. Cardiac symptoms of bradycardia and hypotension can appear early in the course and may require urgent medical attention. Gastrointestinal symptoms usually resolve in 2-4 days but neurological symptoms can last for days to weeks to months. The frequency of symptoms can vary depending on the source of the fish [4]. Recurrence of symptoms following weeks and months of initial poisoning have been reported with certain triggers, namely caffeine, alcohol, any fish, pork, chicken, nuts, excessive physical exertion, and dehydration. This is likely due to the cumulative exposure process, neurological sensitization, or mobilization of toxins from the fat cells [5]. This recurrence imposes a significant morbidity risk and poor quality of life.

Diagnosis

A detailed history can help in the early diagnosis of ciguatera fish poisoning-related illness. History of fish consumption, especially fish from endemic region, a timeline of signs and symptoms, ruling out other common gastrointestinal and neurological conditions. Currently, there is no human biomarker testing available to confirm CFP. Testing uncooked or cooked fish for ciguatoxin remains the gold standard diagnostic tool. Such testing is not readily available and is time-consuming. For example, Florida does not have any fish testing capability and FDOH refers to FDA Gulf Coast Seafood Laboratory in Dauphin Island, Alabama. Commercially available kits are unreliable and not recommended by CDC and FDA.

Reporting

Due to ciguatera fish poisoning-related outbreak risk, any single case should be reported to health authorities. In certain states, such as Florida, reporting of CFP is mandatory. As per chapter 64D-3, Florida Administrative Code, it is required by law for health care providers to report to the county health department within one working day of diagnosis. CFP poisoning and related illness can be reported to FDA via email, .

Treatment

The mainstay of treatment for CFP remains supportive and symptom control. This disease has rarely been reported to be life-threatening. Cardiac manifestation of bradycardia and hypotension should trigger immediate medical attention and hospitalization. Hemodynamic support and ruling out any other potential causes of cardiovascular symptoms should be prioritized. Most patients respond to oral or intravenous hydration, correction of electrolytes, antinausea and antidiarrheal medications. Acetaminophen and non-steroid anti-inflammatory agents can help achieve analgesia.

Intravenous mannitol is the most studied therapy for the treatment of CFP. In 1988, Palafox et al. first described the benefit of mannitol in 24 patients with acute CFP [8]. Since then several other uncontrolled case series and case reports have shown the benefits of intravenous mannitol [9]. Mannitol is an osmotic diuretic and is thought to be helpful by reducing neuronal edema caused by ciguatoxin or potentially a scavenger effect. The optimal dose is described as 1.0 g/kg over 30-45 minutes [10]. While some case reports have shown benefit with a lower dose of 0.5-1 g/kg, administered over 3-4 hours. It can help reduce symptoms and shorten the duration of symptoms, especially if given in 48-82 hours of ingestion of fish [6]. The patient should be well hydrated and electrolyte levels should be optimized before infusion of mannitol. A small double-blind randomized controlled trial (RCT) on 50 patients showed that mannitol was not superior to normal saline in relieving symptoms of CFP but had more side effects [11].

Supportive therapy for chronic neurological symptoms with gabapentin [12], amitriptyline [13,14], and pregabalin [15] have been shown in case reports and case series. Dietary restrictions on various trigger food should be practiced to avoid recurrence of symptoms at least for 3-6 months [6].

## Conclusions

Ciguatera fish poisoning remains prevalent worldwide and continues to be the most common cause of seafood toxin-induced illness. A wide range of symptoms with varied timelines and severity, lack of specific diagnostic tests for human exposure, imposes a significant diagnostic challenge on clinicians. We need better epidemiological surveillance, quick and reliable tests to detect ciguatoxin in fishes, and human markers to identify exposure. At this time, we lack enough sufficient randomized controlled trials to show statistically significant benefits of mannitol therapy.

## References

[1] Friedman MA, Fleming LE, Fernandez M (2008). Ciguatera fish poisoning: treatment, prevention and management. Mar Drugs.

[2] (2022). Ciguatera fish poisoning (CFP). https://www.fao.org/3/y5486e/y5486e0q.htm.

[3] Radke EG, Reich A, Morris JG Jr (2015). Epidemiology of ciguatera in Florida. Am J Trop Med Hyg.

[4] Crump JA, McLay CL, Chambers ST (1999). Ciguatera fish poisoning. Postgrad Med J.

[5] Abraham A, Jester E, Granade HR, Plakas SM, Dickey RW (2012). Caribbean ciguatoxin profile in raw and cooked fish implicated in ciguatera. Food Chem.

[6] Friedman MA, Fernandez M, Backer LC (2017). An updated review of ciguatera fish poisoning: clinical, epidemiological, environmental, and public health management. Mar Drugs.

[7] Morin E, Gatti C, Bambridge T, Chinain M (2016). Ciguatera fish poisoning: Incidence, health costs and risk perception on Moorea Island (Society archipelago, French Polynesia). Harmful Algae.

[8] Palafox NA, Jain LG, Pinano AZ, Gulick TM, Williams RK, Schatz IJ (1988). Successful treatment of ciguatera fish poisoning with intravenous mannitol. JAMA.

[9] Mullins ME, Hoffman RS (2017). Is mannitol the treatment of choice for patients with ciguatera fish poisoning?. Clin Toxicol (Phila).

[10] Pearn JH, Lewis RJ, Ruff T (1989). Ciguatera and mannitol: experience with a new treatment regimen. Med J Aust.

[11] Schnorf H, Taurarii M, Cundy T (2002). Ciguatera fish poisoning: a double-blind randomized trial of mannitol therapy. Neurology.

[12] Perez CM, Vasquez PA, Perret CF (2001). Treatment of ciguatera poisoning with gabapentin. N Engl J Med.

[13] Davis RT, Villar LA (1986). Symptomatic improvement with amitriptyline in ciguatera fish poisoning. N Engl J Med.

[14] Calvert GM, Hryhorczuk DO, Leikin JB (1987). Treatment of ciguatera fish poisoning with amitriptyline and nifedipine. J Toxicol Clin Toxicol.

[15] Brett J, Murnion B (2015). Pregabalin to treat ciguatera fish poisoning. Clin Toxicol (Phila).

